# Unraveling the microbial puzzle: exploring the intricate role of gut microbiota in endometriosis pathogenesis

**DOI:** 10.3389/fcimb.2024.1328419

**Published:** 2024-02-16

**Authors:** Fan Tang, Mengqi Deng, Chunyu Xu, Ruiye Yang, Xuechao Ji, Menglin Hao, Yixiao Wang, Ming Tian, Yuning Geng, Jinwei Miao

**Affiliations:** Department of Gynecologic Oncology, Beijing Obstetrics and Gynecology Hospital, Capital Medical University, Beijing Maternal and Child Health Care Hospital, Beijing, China

**Keywords:** endometriosis, gut microbiota, immune, lipopolysaccharide, inflammatory

## Abstract

Endometriosis (EMs) is a prevalent gynecological disorder characterized by the growth of uterine tissue outside the uterine cavity, causing debilitating symptoms and infertility. Despite its prevalence, the exact mechanisms behind EMs development remain incompletely understood. This article presents a comprehensive overview of the relationship between gut microbiota imbalance and EMs pathogenesis. Recent research indicates that gut microbiota plays a pivotal role in various aspects of EMs, including immune regulation, generation of inflammatory factors, angiopoietin release, hormonal regulation, and endotoxin production. Dysbiosis of gut microbiota can disrupt immune responses, leading to inflammation and impaired immune clearance of endometrial fragments, resulting in the development of endometriotic lesions. The dysregulated microbiota can contribute to the release of lipopolysaccharide (LPS), triggering chronic inflammation and promoting ectopic endometrial adhesion, invasion, and angiogenesis. Furthermore, gut microbiota involvement in estrogen metabolism affects estrogen levels, which are directly related to EMs development. The review also highlights the potential of gut microbiota as a diagnostic tool and therapeutic target for EMs. Interventions such as fecal microbiota transplantation (FMT) and the use of gut microbiota preparations have demonstrated promising effects in reducing EMs symptoms. Despite the progress made, further research is needed to unravel the intricate interactions between gut microbiota and EMs, paving the way for more effective prevention and treatment strategies for this challenging condition.

## 1 Introduction

Endometriosis (EMs) is a common gynecological disorder where the normal glandular and stromal tissue of the uterus grows outside the uterine cavity, causing symptoms such as progressive dysmenorrhea, dyspareunia, chronic pelvic inflammation, and infertility. EMs is most commonly diagnosed in women aged 25-45, with a global prevalence of approximately 10%-15%, and the highest incidence observed in Asian women (Smolarz et al., 2021).

Despite decades of research, its exact pathogenesis is still not well understood. The main pathophysiological theories include the implantation theory, retrograde menstruation theory, metaplastic theory, and genetic expression differences theory. Among them, the most well-accepted hypothesis is based on retrograde menstruation, which proposes that endometrial tissue fragments shed during menstruation and flow back through the fallopian tubes and implant in the pelvic cavity, leading to the formation of endometriotic lesions. However, it is estimated that only 10% of women with retrograde menstruation develop EMs, indicating that this theory cannot fully explain the pathogenesis of the disease (Ahn et al., 2015b; Laschke and Menger, 2016). Recent studies suggest that although EMs is a benign condition, some of its biological characteristics, such as infiltration, migration, and recurrence, are similar to malignant tumors. The “Eutopic endocardium determinism” theory has been considered a breakthrough to supplement the retrograde flow theory, which suggests that mutations in certain determinants of endometrial tissue may contribute to stronger angiogenesis, migratory and invasive ability, leading to the development of EMs. This theory has explained to a certain extent the phenomenon mentioned above.

EMs lesions mainly occur in the pelvic cavity, which is also a container for holding the small intestine and colorectum. The intestine contains a large number of gut microbiota, which exert an important role in maintaining pelvic stability (Rahman-Enyart et al., 2021). Several studies have confirmed the idea that gut microbiota is involved in many inflammatory, immune, and proliferative diseases (Chadchan et al., 2021; Jiang et al., 2021; Shan et al., 2021). Similarities between EMs and irritable bowel syndrome(IBS) or inflammatory bowel disease(IBD) include recurrent abdominal pain, cramping, anxiety, and a local inflammatory microenvironment in lesions (Peters, 2022). Indeed, patients with EMs in a large-scale study had a 50% increased risk of inflammatory bowel disease compared with the general population (Chiaffarino et al., 2020). Around 20% of endometriosis patients also present with symptoms indicative of IBS (Salmeri et al., 2023b). Growing evidence has shown dysbiosis is involved in the occurrence, development, and aggravation IBD and IBS (Inczefi et al., 2022; Salmeri et al., 2023b), and there is a similar link between gut dysbiosis and the pathogenesis of EMs. In addition, many studies have provided a correlative relationship between EMs and gut microbiota. Therefore, changes in the pelvic environment may contribute to the pathogenesis of EMs, and gut microbiota may be a key regulator in the development of EMs.

This article aims to review the latest research progress and explore the relationship between gut microbiota imbalance and the development of EMs, to provide a theoretical basis and clinical treatment strategies for the management of this disease. At the same time, more research is badly needed to better understand the pathogenesis of EMs and help prevent and treat this condition more effectively.

## 2 Overview of gut microbiota

The gut microbiota refers to a diverse and abundant microbial system in the human intestine, which includes the gastrointestinal microbiota, cyanobacteria, spirochetes, and anaerobic microorganisms (Mańkowska et al., 2022). These microorganisms maintain a stable balance through mutual constraints and also participate in the metabolism and absorption of intestinal nutrients, providing energy and protecting the normal function and immune regulation of the body. However, when the body’s immune system is compromised or when gastrointestinal infections occur, the balance of gut microbiota is disrupted, resulting in a reduction in beneficial bacteria and an increase in pathogenic bacteria, leading to inflammatory reactions and gastrointestinal infections. Moreover, gut microbiota imbalance can also cause diseases such as diabetes, hypertension, colon cancer, allergic reactions, and autoimmune diseases (Gholizadeh et al., 2019). Studies have shown that the proportion of harmful bacteria has increased significantly in the intestines of patients with gut microbiota imbalance and they can release exogenous cytotoxins into the bloodstream, which are significantly correlated with the expression of COX-2 and PGE2 (Biarc et al., 2004). In addition, the complex network relationship between gut microbiota and the enteric nervous system has led to the concept of the microbiota-gut-brain (MGB) axis (Erny et al., 2015), which is a bidirectional regulatory pathway that includes the endocrine system, immune system, autonomic nervous system, gut microbiota metabolism system, and enteric nervous system (Kim and Shin, 2018).

## 3 Gut microbiome in EMs

The gastrointestinal tract is a complex ecosystem consisting of a stable balance of intestinal mucosal cells, immune cells, and microbial communities. Eubiosis is characterized by high levels of Firmicutes and Bacteroidetes (>90%) and a low percentage of Proteobacteria, while dysbiosis is linked to an altered F/B ratio (Qin et al., 2010).

In recent years, with the continuous improvement of gene sequencing technology, more and more studies have uncovered the significant roles of the gut microbiota in the pathogenesis of EMs, albeit with conflicting results. A systematic review published by Leonardi et al. in 2019 found that endometriosis is associated with an increased presence of Proteobacteria, *Enterobacteriaceae*, *Streptococcus*, and *Escherichia coli* across various microbiome sites (Leonardi et al., 2020). In EMs patients, several abnormal gut microbiota have been identified, including *Gardnerella*, *Streptococcus*, *Enterococcus*, and *Escherichia coli*, which are present in higher amounts than in healthy women. In addition, the ratio of *Shigella* and *Escherichia coli* is significantly different in fecal samples from severe EMs patients (Kovács et al., 2021). 16S rRNA sequencing analysis had shown that the alpha and beta diversity of gut microbiota is lower and the abundance of 12 genera, such as *Bacteroides*, *Parabacteroides*, *Clostridium difficile*, *Streptococcus*, and *Gamma Proteobacteria*, was higher in EMs patients compared with those in normal individuals (Svensson et al., 2021). Shan et al. performed 16S rRNA gene sequencing on the gut microbiota of fecal samples of 12 stage III-IV EMs patients and 12 healthy controls, with results showing that the alpha diversity of the gut microbiota in the EMs group was lower than that in the control group, and the ratio of Firmicutes/Bacteroidetes was higher (Shan et al., 2021). The endometriosis and mock mice shared similar alpha diversity gut microbiota (Yuan et al., 2018), and this was also reported in a clinical study from stage 3/4 endometriosis and healthy controls (Ata et al., 2019). The beta diversity index was significantly higher in the endometriosis mice group, compared with controls (Yuan et al., 2018). There were significant differences in the abundance of Actinobacteria, Tannerellaceae, *Blautia*, *Bifidobacterium*, *Dialister*, and *Streptococcus* between the two groups. The human peritoneal microbiome analysis revealed the abundance of *Acidovorax*, *Devosia*, *Methylobacterium*, *Phascolarctobacterium*, and *Streptococcus* in the peritoneal fluid of endometriosis patients were more abundant than the matched controls (Yuan et al., 2022). In EMs rats, the gut microbiota alters with an increase in the ratio of Firmicutes to Bacteroidetes and a decrease in the abundance of Ruminococcaceae, which is closely related to inflammation (Cao et al., 2020). In a recent study, endometriotic lesion growth is reduced by depletion of the gut microbiome, and the feces from mice with endometriosis can aggravate lesion growth, which proved there is a close connection between gut microbiota and endometriosis (Chadchan et al., 2023). These studies demonstrate that there are significant differences in gut microbiome expression between EMs patients and healthy women. The precise alterations in the microbiome related to endometriosis are still under investigation. However, the significance of these changes is supported by the existence of several proposed mechanisms through which the gut microbiota influences endometriosis.

## 4 Possible mechanism of intestinal flora imbalance affecting EMs pathogenesis

### 4.1 The intestinal microbiota is involved in immune-mediated chronic inflammatory regulation in EMs

EMs has been considered as an immune-compromised chronic inflammatory disease, emphasizing the indispensable role of immunological factors in the pathogenesis of EMs (Kvaskoff et al., 2015). Observations of continuous growth of endometrial lesions in ovariectomized animals suggest that the immune system in the pelvic environment may regulate the growth of ectopic lesions (Novella-Maestre et al., 2012). Both local and systemic immune mechanisms favor the growth and maintenance of endometriotic lesions due to imbalanced immune cell populations and altered cytokine profiles (Berkkanoglu et al., 2003; Han et al., 2023). A multicenter case-control study suggests that the presence of concomitant autoimmunity in endometriosis has a significant additive negative impact on embryo implantation (Salmeri et al., 2023a).

The capacity of the intestinal microbiota to shape immune responses outside of the intestine is well documented (Kau et al., 2011). Studies have highlighted the ability of the microbiota and specifically *segmented filamentous bacteria* to support the development of autoimmune arthritis (Wu et al., 2010) and experimental allergic encephalomyelitis (Lee et al., 2011), both of which have been linked to excessive Th17 responses. Disturbance of gut microbes of the mucosal immune system or “dysbiosis” affects normal physiological function (Allaire et al., 2018) with implications for inflammatory disease (Islam et al., 2018). Dysbiotic bacteria can digest the intestinal protective mucus layer and interact directly with enterocytes, typically leading to an increase in local and systemic inflammation (Blander et al., 2017). Studies suggest that metabolites and endotoxins produced by the intestinal microbiota can increase intestinal mucosal permeability, and ultimately lead to weakened intestinal barrier function and increased intestinal mucosal permeability, a condition known as “leaky gut”. This allows various inflammatory factors and toxic substances to enter the bloodstream and trigger antigen-antibody binding and immune reactions (Khan et al., 2017). Mohling et al. investigated whether patients with laparoscopically confirmed endometriosis exhibit higher rates of impaired intestinal permeability compared to healthy controls and pelvic pain patients without endometriosis. Out of 20 patients with laparoscopically defined endometriosis, 45% had impaired intestinal permeability, whereas none of the 9 patients without endometriosis (control subjects) showed impairment (*P*=0.027). The study suggests a potential association between impaired intestinal permeability and endometriosis, emphasizing the need for further research to understand its role in the pathogenesis and potential diagnostic implications for endometriosis (Mohling et al., 2023). Xholli et al. explored the role of zonulin, a protein responsible for regulating intestinal permeability, which could help elucidate the presence of gastrointestinal symptoms in endometriosis patients (Xholli et al., 2023).

#### 4.1.1 Immune cells

##### 4.1.1.1 Macrophages

Depending on activation state and surface markers, macrophages are classified as ‘classically activated’(M1) or ‘alternatively activated’(M2). M1 secrete pro-inflammatory factors, IL-12, IL-23, and NOS, which activate T helper 1(Th1) T cells and lead to a pro-inflammatory cascade. Whereas M2 is involved in angiogenesis, coordination of tissue repair, and production of IL-10, which leads to an immunosuppressive phenotype and activation of Th2 cells (Ning et al., 2016).

Early and active lesions of pelvic endometriosis and their adjacent peritoneum harbor abundant macrophages involved in the growth of endometriosis (Khan et al., 2004). The concentration and proportion of macrophages in the ascites of patients with endometriosis were significantly increased, with an enhanced M2:M1 ratio (Zou et al., 2021). M2 predominating in lesions and peritoneal fluid may contribute to pain experienced by women with endometriosis by promoting nerve fiber growth (Bacci et al., 2009). Macrophages in the peritoneal fluid of women with endometriosis exhibit activation of the NF-kB pathway (Lousse et al., 2008). Ectopic endometrial cells may escape removal by macrophages with reduced phagocytic ability (Liu et al., 2019). The fibrogenesis ability and decreased phagocytotic ability of macrophages contribute to endometriotic fibrotic foci formation and lesion proliferation (Duan et al., 2018). The predominance of the endometrial M1 pro-inflammatory phenotype and pro-inflammatory cytokine secretion provides an inhospitable environment for pregnancy.

The number of macrophages and the concentration of peritoneal IL-1β, TNF-α, IL-6, and TGF-β1 is lower in mice endometriotic lesions treated with broad-spectrum antibiotics like ampicillin or metronidazole compared with that in vehicle-treated mice (Chadchan et al., 2019; Jeljeli et al., 2020). *Escherichia coli*-derived endotoxin-induced macrophage- and TLR4-mediated higher pro-inflammatory reactions in the pelvis of women with endometriosis (Khan et al., 2010). Gut dysbiosis may lead to abnormal β-glucuronidase secretion, promoting M0 to M2 polarization affecting endometrial stromal cell proliferation, invasion, and migration, as well as induced macrophage infiltration and development of endometriotic lesions in the EMs mouse model (Wei et al., 2023).

##### 4.1.1.2 Neutrophils

The number of neutrophils is increased in the peritoneal cavity of women with endometriosis. In addition, neutrophil extracellular traps are increased in the peritoneal fluid of women with endometriosis (Berkes et al., 2014). Angiogenic factors such as VEGF and pro-inflammatory cytokines, including IL-8 and CXCL10, and also reactive oxygen species produced by neutrophils may increase the number of endometriotic lesions to promote disease progression (Lin et al., 2006; Takamura et al., 2016).

##### 4.1.1.3 CD4+ T cells

Upon stimulation, naive CD4+ T cells can differentiate into four major subtypes: T helper 1 (Th1), Th2, Th17, or regulatory T cell (Treg). These various CD4+ T cell subtypes are distinguished by their expression of various transcription factors and cytokines. Th1, Th2, Th17, and Treg cells are increased in peritoneal fluid and blood of endometriosis compared to controls (Podgaec et al., 2007; Li et al., 2014; Gogacz et al., 2016). In endometriotic lesions, CD4+ Th1 is decreased but Treg is increased compared to eutopic endometriosis endometrium (Olkowska-Truchanowicz et al., 2013; Takamura et al., 2015). The percentage of Th17 cells in the pelvic peritoneum of endometriotic patients with stage III/IV endometriosis was higher than that in patients with stage I/II endometriosis (Gogacz et al., 2016). IL-17 is an immune regulatory factor mainly produced by Th17 cells, which could stimulate the secretion of angiogenic factors and pro-inflammatory cytokines, accelerating the establishment and growth of ectopic lesions (Ahn et al., 2015a). The number of Treg cells significantly increased in the peritoneal lesions of patients with ovarian endometrioma compared with patients without endometriosis (Khan et al., 2019). A large of studies confirmed that Treg cells suppress the immune response and promote the progression of endometriosis (Basta et al., 2014; Tanaka et al., 2017; Olkowska-Truchanowicz et al., 2021).

Chadchan, S.B. et al. showed that there are a lower number of immune cell populations such as M2-like macrophage, CD19+ B cells, total T cells, CD4+ T cells, and CD8+ T cells, and smaller endometriotic lesions in the peritoneum of microbiota-depleted mice compared to the control group (Chadchan et al., 2023). The *Firmicutes* and *Clostridium* species in the intestinal microbiota metabolize to produce butyrate, while *Bifidobacterium* and *Actinobacteria* produce acetate. Butyrate can promote the differentiation of primary T cells into regulatory T cells (Tregs) and directly regulate T cell responses. Acetate and butyrate can regulate the interaction of dendritic cells and T cell complexes (DC-T) by inhibiting the expression of nuclear factor κB through histone deacetylase inhibitors (HDACi) and inducing the transcription of anti-inflammatory genes, leading to the differentiation of Tregs and the maintenance of immune balance (Kedmi et al., 2022).

Gut microbiota such as *segmented filamentous bacteria* and *Clostridia* can participate in the differentiation of Th17 cells and promote the induction, migration, and proliferation of Treg cells (Wu et al., 2010; Goto et al., 2014). Gut-residing *segmented filamentous bacteria* induce an increase in the number of arthritogenic or encephalitogenic Th17 cells, resulting in exacerbation of arthritis and experimental autoimmune encephalomyelitis (Lee et al., 2011; Liu et al., 2020). *Polymorphic rod-shaped bacteria* and *Firmicutes* have been found to significantly increase in colon cancer tissue, and their metabolites can regulate intestinal immune function, including the differentiation of Th17 and Treg cells (Cong et al., 2022). IL-37 is a natural anti-inflammatory cytokine that participates in the regulation of gut microbiota and immune response. Dysbiosis of gut microbiota can increase the expression of IL-37, recruit neutrophils and natural killer cells in the colonic lamina propria and mesenteric lymph nodes, cause damage to the intestinal epithelial barrier, and increase inflammatory responses and immune dysfunction (Zhang et al., 2010).

Further research has revealed the overexpression of programmed death receptor-1 (PD-1) and programmed death ligand-1 (PD-L1) on the surfaces of these immune cells (Dai et al., 2014). On the surface of normal immune cells, the expression of PD-1 and PD-L1 is low. However, the PD-1/PD-L1 signaling pathway is overactivated accompanied by stimulated inflammation, which inhibits the activation and proliferation of T cells in the local inflammatory microenvironment. At the same time, the cytotoxic effect of T cells on abnormal cells have been greatly reduced, leading to immune tolerance and subsequently reducing the body’s immunity, resulting in immune escape (Vallvé-Juanico et al., 2019). Therefore, the sustained stimulation and activation of the PD-1 pathway by a large amount of bacterial endotoxin caused by dysbiosis of gut microbiota leads to overexpression of PD-1 and PD-L1, which induces exhaustion of immune T cells and immune escape.

#### 4.1.2 Inflammatory mediators

It is well-established that peritoneal inflammation, attributable to the high local cytokine concentration, is a hallmark of endometriosis(Allaire et al., 2023). Several key inflammatory mediators, including COX-2, IL-1β, IL-8, tumor necrosis factor (TNF)-α, PGE2, and E2, are elevated in endometriotic lesions compared with eutopic endometrium. Increased anti-inflammatory cytokines, such as IL-6, IL-10, IL-15, and TGF-β in the peritoneal fluid may mitigate the pro-inflammatory effects of PGE2 and NF-κB (Wang et al., 2018). The binding between lipopolysaccharide and TLR-4 significantly increases the concentration of peritoneal cavity immune cells, especially macrophages (Emani et al., 2015), which produce TNF-alpha, IL-1 receptor, vascular endothelial growth factor (VEGF), IL-6, IL-8, and IL-17, and which can promote the formation, infiltration, and neoangiogenesis of endometriotic peritoneal nodules (Khan et al., 2010; Khan et al., 2018). In 2021, Jiang et al. hypothesizes that dysbiosis lead to the elevation of proinflammatory cytokines compromising the immunosurveillance, creating an environment that maintain the vicious cycle of endometriosis onset and progression (Jiang et al., 2021).

#### 4.1.3 Angiogenesis-related substances

In women with endometriosis, there are a large number of neovascularizations around the ectopic lesions in the abdomen, and vascular formation is an important factor in ectopic lesion adhesion, proliferation, and repeated bleeding. The adhesion-invasion-vascularization process is required for ectopic endometrium to grow in the abdomen.

Vascular endothelial growth factor (VEGF) is the main regulator of vascularization, promoting endothelial cell differentiation, proliferation, migration, and inducing gut inflammation. Coordinated efforts by both M1 and M2 macrophages are required for angiogenesis and scaffold vascularization (Spiller et al., 2014).

The angiopoietin (ANG) protein family is associated with angiogenesis, upregulated in various cancers (Miyake et al., 2015). ANG is involved in hypoxia-induced angiogenesis in endometriosis and the expression of ANG in endometriotic tissue is upregulated (Fu et al., 2018). ANG plays an important role in regulating gut microbiota balance and inhibiting inflammation. There is an ANG-microbiota axis in the gut, and ANG can regulate gut microbiota in the form of antimicrobial peptides. In mice with dysbiosis of gut microbiota, the absence of ANG leads to a decrease in *Helicobacter* species but induces an inflammatory response when the α-Enterobacteriaceae strains in the colon increase (Korecka et al., 2013; Carbone et al., 2018; Sun et al., 2021). Ang4, induced by *Bacteroides thetaiotaomicron*, influences gut microbial ecology and shape innate immunity. Mouse Ang1 and human angiogenin, circulating proteins induced during inflammation, exhibit microbicidal activity contributing to systemic responses to infection (Hooper et al., 2003). Whether the ANG-microbiota axis contributes to the pathogenesis of endometriosis requires further investigation.

#### 4.1.4 Metabolites

Intestinal microbiota breaks down excess polysaccharides in the gut into short-chain fatty acids (SCFAs). SCFAs such as acetate, propionate, n-butyrate, pentanoic (valeric) acid, and hexanoic (caproic) acid are used as an energy source by enterocytes or are transported into the bloodstream, which functions as protecting the gut mucosal barrier, regulating metabolism and immune function (den Besten et al., 2013). Feces from mice with endometriosis contained less of SCFAs and n-butyrate inhibited human endometriotic cell survival and lesion growth through G-protein–coupled receptors, histone deacetylases, and a GTPase activating protein, RAP1GAP (Chadchan et al., 2021). *In vitro* studies have shown that SCFAs can inhibit the activation of TLR4 signaling pathways, inhibit the secretion of pro-inflammatory cytokines, and reduce gut inflammation (Kim, 2023). The concentration of butyrate in the lumen is positively correlated with the number of Tregs (Arpaia et al., 2013). Butyrate can modify the cytokine production profile of helper T cells and promote intestinal epithelial barrier integrity, which in turn can help limit exposure of the mucosal immune system to luminal microbes and prevent aberrant inflammatory responses (Kau et al., 2011). Butyrate and propionate can block the production of dendritic cells by influencing specific transcription factors of dendritic cell precursors but do not affect granulocyte production (Singh et al., 2010). Future studies are needed to determine how SCFAs influence ectopic endometrial implantation and propagation by regulating immune response.

Arpaia et al. found that butyrate is essential for extrathymic but dispensable for thymic Treg-cell differentiation (Arpaia et al., 2013). Tregs are mainly produced in the thymus from where they migrate to the circulation as natural Tregs(nTregs), and a much smaller subpopulation differentiates in the periphery from naïve T cells into induced Tregs(iTregs) (Tanoue et al., 2016). It was postulated that peripheral regulatory T-cell changes induced by decreased butyrate may not influence the establishment of an anti-inflammatory environment by suppressing the activation of the immune system evoked by the endometriotic foci. Loss of balance between Th1/Th2/Th17 and Tregs leads to inappropriate secretion of T-cell-related cytokines and inflammation that induces the progression of endometriotic lesions (Szukiewicz et al., 2022).

In summary, the metabolites of gut microbiota may play a crucial role in intestinal immune function, while more in-depth functional studies are warranted to uncover the precise mechanism of specific immune cells influenced by gut microbiota. Imbalance of gut microbiota may lead to inflammation and immune dysfunction, thus regulating the balance of gut microbiota is one of the important strategies for the prevention and treatment of gut-related diseases.

### 4.2 The intestinal microbiota affects the generation of serum LPS and participates in the pathogenesis of EMs

Dysbiosis of the intestinal microbiota can lead to an increase in Gram-negative bacteria, causing a large amount of LPS to enter the circulatory system inducing chronic inflammation (He et al., 2019). A study of macaques found that there are significant changes in the fecal bacteria of the EMs group compared to the control group, showing a decrease in bifidobacteria and an increase in Gram-negative bacteria. Moreover, the incidence of intestinal inflammation was higher in the EMs macaques group than in the control group (Bailey and Coe, 2002).

LPS is an important component of the outer membrane of Gram-negative bacteria, which normally exists in various parts of the human body such as the skin, oral cavity, and gastrointestinal tract. However, when the level of LPS increases, it can cause a large amount of growth and reproduction of intestinal pathogens while inhibiting the activity of beneficial bacteria (Maldonado et al., 2016). LPS stimulates the endometrial stromal cells to produce a large amount of tumor necrosis factor-alpha (TNF-α) and IL-8, and promotes the mitotic activity of human endometrial stromal cells (Khan et al., 2010). At the same time, the expression of COX-2 and PGE2 is upregulated, promoting the proliferation and invasion of human endometrial stromal cells (Iba et al., 2004).

LPS induces the production of inflammatory factors and vascular endothelial growth factors, allowing the refluxed endometrial fragments to implant and form ectopic lesions in the abdominal cavity (Matsuzaki et al., 2020). LPS can promote ectopic endometrial adhesion and invasion by inducing the expression of adhesion molecules between endometrial and pelvic peritoneal cells (Keyama et al., 2019). Chenodeoxycholic acid (CDCA), a component of the secondary bile acid biosynthesis, increases in the intestine of EMs mice (Ni et al., 2020). It is closely related to gut microbiota and contributes to promoting intestinal homeostasis. CDCA blocks LPS-induced activation of the myosin light chain kinase pathway, thereby protecting against the LPS-induced impairment of the intestinal epithelial barrier function (Gadaleta et al., 2011; Song et al., 2019).

Epithelial-mesenchymal transition (EMT) also plays an important role in the adhesion and invasion of ectopic epithelium in EMs, and it is also an important factor for successful implantation and lesion migration of ectopic epithelium (Xiong et al., 2015; Xiong et al., 2016). LPS upregulates TLR4 expression, induces EMT phenotype, and contributes to the invasion of ectopic endometrium (Ying et al., 2018, 4). TLR4 is a type I transmembrane protein that plays an important role in innate immunity. *In vitro* cell and animal experiments have confirmed that the intestinal microbiota LPS-TLR4 pathway is involved in various inflammatory bowel diseases. As the main type of TLR, TLR4 is a natural immune receptor that mediates LPS response and can be recognized by LPS receptors in the cell wall of Gram-negative bacteria. After binding to LPS, TLR4 can induce an inflammatory cascade reaction, causing the release of a large number of inflammatory mediators, thereby causing inflammatory damage to the digestive tract. The inflammatory reaction involving the intestinal microbiota LPS-TLR4 pathway is mainly caused by *Bacteroides*, and patients with dysbiosis of the intestinal microbiota are accompanied by elevated levels of peripheral blood monocyte TLR4 and peripheral blood inflammation (Li et al., 2021).

In women with endometriosis, the body is in a state of low inflammation. When the bile acid receptor 1 on white blood cells is activated, their phagocytic ability decreases, while LPS-induced pro-inflammatory cytokines such as TNF-α, IL-1α, IL-1β, and IL-6 are inhibited. The downregulation of LPS-induced TNF-α expression inhibits macrophage inflammatory responses (Peng et al., 2020). On the other hand, TLR4 forms a dimer with leukocyte differentiation antigen 14, activating downstream MyD88 and TIR domain-containing adaptor protein signaling pathways to upregulate the expression of pro-inflammatory cytokines, chemokines, and interferons, exacerbating gut inflammation (Li et al., 2020).

When the gut microbiota is imbalanced, the proportion of tight junction proteins and occludin proteins between the host intestinal epithelial cells decreases, increasing intestinal mucosal permeability. Gut microbiota metabolizes LPS, which enters the bloodstream and binds to lipopolysaccharide-binding protein (LBP). LBP then activates the receptor CD14 on the surface of immune cells, which helps to recognize and activate TLR4, thereby activating the MyD88/NF-κB signaling pathway and promoting the release of IL-1, IL-6, TNF-α, and other inflammatory mediators. This leads to a cascade of inflammatory reactions in the body, inducing EMs and placing the body in a state of low-grade inflammation (Płóciennikowska et al., 2015).

### 4.3 Gut microbiota involve in hormonal regulation

High levels of estrogen can induce proliferative diseases such as EMs, uterine fibroids, and endometrial cancer by stimulating the proliferation of female reproductive tract epithelial cells (Somasundaram et al., 2020). Intestinal bacteria play an important role in estrogen metabolism, evidenced by the observation that estrogen levels are reduced because of the use of antibiotics (Chadchan et al., 2019). Also the gut bacterial species and proportion altered in many sex hormone-driven cancers, such as endometrial, prostate, and breast cancer (Sepich-Poore et al., 2021).

Gut microbiota participates in the estrogen cycle, forming the estrogen-gut microbiota axis. Gut microbiota including *Firmicutes*, *Bacteroidetes*, and *Bifidobacterium* have genes related to glucuronidase activity, thus could be indicative of an altered astrobleme and dysregulated estrogen metabolism in mice with endometriosis compared to controls (Pradhan et al., 2016; Baker et al., 2017). Ecological imbalance increases circulating estrogen levels, stimulates ectopic endometrial invasion and growth, and is accompanied by cyclic bleeding and pain. The β-glucuronidase secreted by gut microbiota participates in estrogen regulation by metabolizing estrogen from a bound form to an unbound form, which is then reabsorbed and involved in the regulation of circulating estrogen levels through enterohepatic circulation (Junkka and Ohlsson, 2023). β-glucuronidase can affect the growth of hormone-dependent tumors in the body by participating in intestinal estrogen metabolism (Baker et al., 2017). It is suspected that increased β-glucuronidase-producing bacteria in the gut of endometriosis patients leads to an increase in circulating estrogen levels, disrupting the balance between circulating estrogen levels and gut microbiota.

Estrogen metabolism analysis of EMs patients shows that there are significant differences in the expression of 17β-estradiol, 16-keto-17β-estradiol, 2-hydroxyestrone, and 2-hydroxyestradiol compared to healthy individuals, and gut microbiota of EMs patients are positively correlated with urinary estrogen (Ser et al., 2023). In addition, bacteria such as the genus Bacteroides in the gut express 17β-dehydrogenase, which can decompose testosterone into androstenedione, demonstrating the involvement of gut microbiota in sex hormone synthesis and metabolism (Baker et al., 2017; Le et al., 2021).

The gut microbiota are equally important for the metabolism and circulation of androgens. One study measured the level of non-glucuronidated testosterone in mice and found that the distal colon of germ-free mice showed high levels of glucuronidated testosterone and 5α-dihydrotestosterone, but significantly lower levels of free dihydrotestosterone, suggesting that gut microbiota affect intestinal androgen metabolism and glucuronidation of dihydrotestosterone (Shin et al., 2019). Therefore, gut microbiota plays an important regulatory role in the biotransformation of estrogen, which can affect hormonal balance.

## 5 Mouse models used to address relationship between gut microbiota dysbiosis and endometriosis

Manipulation of gut microbiota in animal models constitutes a key experimental approach to demonstrate causality between gut microbiota dysbiosis and the occurrence of a given disease. In particular, mouse models have been increasingly used over the last years to address the causal role of microbiota in endometriosis, getting further insights into the role of these microorganisms in the occurrence or chronicity of endometriosis.

Germ-free mice generated by surgically delivering pups, sterilizing them, and rearing them in germ-free isolators, which are considered as the gold standard to study the effect of the complete absence of microbes, to establish mice with precisely defined microbiota composition or to perform intestinal microbiota transfer experiments. However, surgically inducing endometriosis in germfree isolators is extremely challenging and germ-free mice lack an educated immune system with several developmental defects (Al-Asmakh and Zadjali, 2015).

Microbiota-depleted (MD) mice are generated by raising mice under standard conditions and treated with broad-spectrum antibiotics. Antibiotics provided in drinking water or via oral gavage t. However, administration of broad-spectrum antibiotics in drinking water may increase baseline morbidity and mortality in mice (Hill et al., 2010).

The limited microbiota in laboratory mice is a growing concern in human immunology and clinical research. As wild mice live in natural habitats similar to humans, they had higher proportions of effector and memory T cells and higher cytokine production, whereas laboratory mice lacked memory CD8+ T cell subsets that experienced protection against pathogen invasion (Beura et al., 2016). Utilizing the wild mice microbiome could be an option to investigate immunological properties. It has been reported that the rewilded laboratory mouse via wild microbiota colonization showed similar microbial community and immune system fitness (Rosshart et al., 2019). Therefore, the wildling or humanized model by fecal transfer would improve the value of preclinical findings.

Many parameters should thus be considered by investigators before selecting one of these protocols.

## 6 The prospects of gut microbiota for the diagnosis and treatment of endometriosis

With the continuous development of gene sequencing technology, it has become increasingly clear that the gut microbiota plays an important role in the pathogenesis of EMs. Researchers have found significant differences in gut microbiota between EMs patients and healthy individuals, among which the genus *Streptococcus* is considered a potential biomarker for EMs patients. Another study indicated that the depletion of *Lachnospiraceae Ruminococcus* in the gut might be a biomarker for endometriosis. Four differential metabolites, namely chenodeoxycholic acid (CDCA), ursodeoxycholic acid (UDCA), ALA, and 12,13s-epoxy-9z,11,15zoctadecatrienoic acid (12,13-EOTrE) are found through fecal metabolomics and gut microbiota research of the EMs animal model, suggesting their potential as important biological indicators to distinguish the disease (Ni et al., 2020). Research has shown that the abundance of Bacteroides in the feces of EMs mice is higher than that of normal mice. After treatment with metronidazole, Bacteroides was not detected in the feces of EMs mice, and the ectopic lesions decreased in size (Chadchan et al., 2019). Subsequently, gavage with fecal bacteria from EMs mice previously treated with metronidazole significantly reduced the size of ectopic endothelial lesions, indicating that the development of EMs can be weakened by antibiotics.

Clinical animal experiments have shown that gut microbiota preparations have achieved certain therapeutic effects in the treatment of EMs, among which fecal microbiota transplantation (FMT) is considered an important way to treat gut dysbiosis in EMs. FMT therapy mainly involves the infusion of healthy donor fecal suspensions into recipients to regulate gut microbiota dysbiosis and treat EMs.

In addition, gut microbiota can produce butyrate, which activates the protein Rap1GAP through GPCR, HDAC, and Rap1 GTPase to inhibit the survival and growth of endometrial ectopic cells. Studies have shown that butyrate treatment in an EMs mouse model can reduce ectopic endometrial lesions, providing some insights for clinical treatment. Therefore, the use of gut microbiota preparations for the diagnosis and treatment of EMs has research prospects, but further exploration and improvement are still needed.

## 7 Limitations and future directions

**Figure 1:**
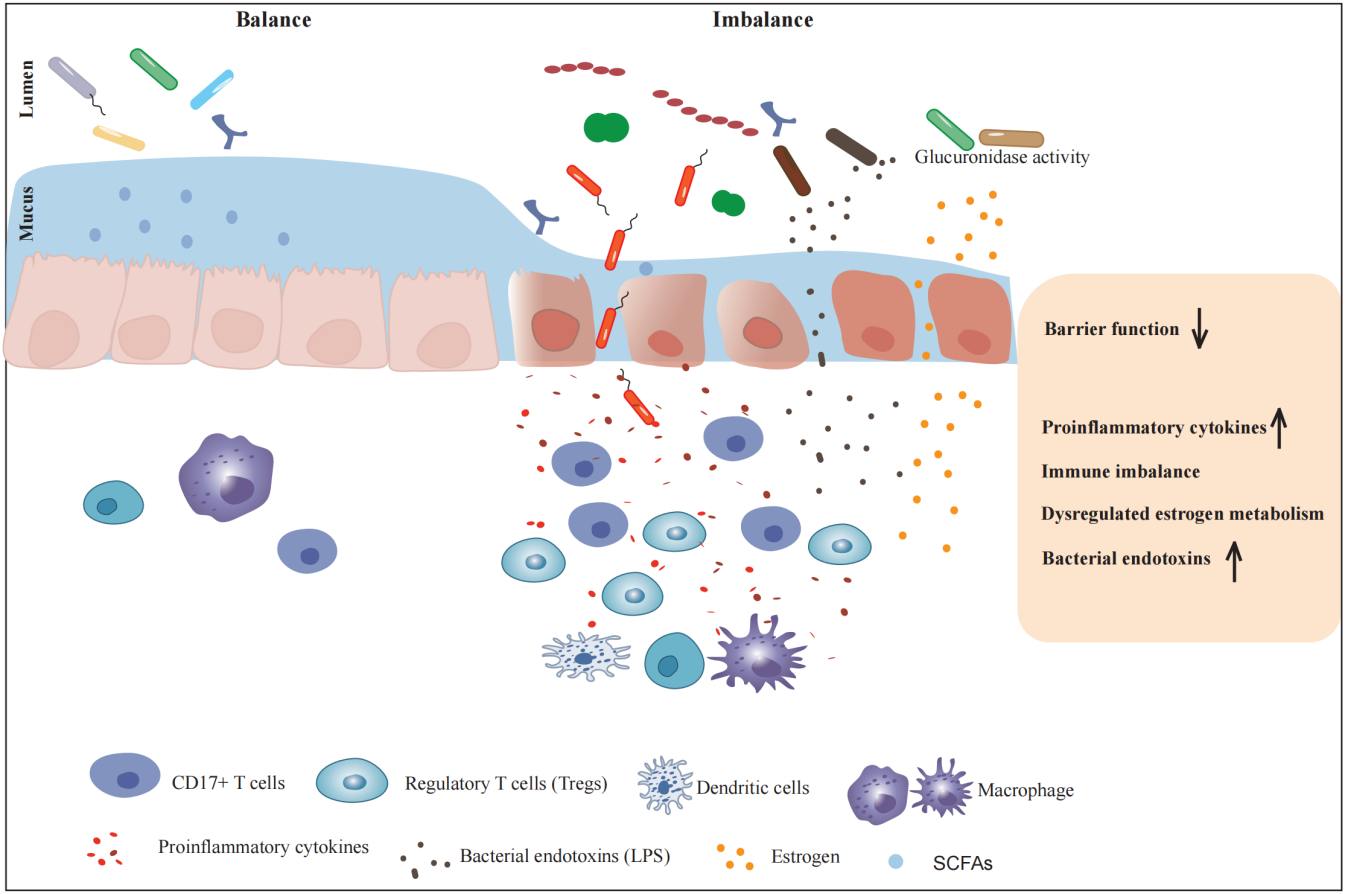
The influence of gut microbiota on the pathogenesis of endometriosis. Metabolites and endotoxins produced by the intestinal microbiota can lead to weakened intestinal barrier function and increased intestinal mucosal permeability. Multiple aspects such as immune system regulation, release of inflammatory factors, angiogenesis-related substances involvement, hormonal regulation, and endotoxin production are comprehensively involved in creating a favorable environment for the occurrence and development of endometriosis.

In summary, endometriosis (EMs) is a common gynecological disease that seriously affects the physical and mental health of female patients due to its adhesive, invasive, and recurrent characteristics. The relationship between EMs and gut microbiota imbalance is under-studied, intestinal microbiota may participate in the pathogenesis of EMs through immune regulation, LPS generation, pro-inflammatory cytokine, and ANG release, and other mechanisms **Figure 1**. Clinical studies have shown that intestinal microbiota preparations also have certain efficacy in the treatment of EMs, providing a research direction and theoretical basis for the diagnosis and treatment of EMs patients with intestinal microbiota. Upper genital tract and gut microbiota might be cofactors causing the development and growth of endometriosis, only the latter has been elaborated on this review. However, the present explanations are speculations arising from interesting observations, but there is a paucity of robust studies to demonstrate causal relationships. Future efforts could explore the role of particular microbiota or derived metabolites on immune cells or inflammatory mediators in endometriosis patients. In-depth functional studies with specific immune cell-deficient mouse models will uncover the precise mechanism by which gut microbiota drives peritoneal immune function in endometriosis. Since intestinal microbiota are numerous and diverse and play important roles in various systems of the human body, robust studies that employ rigorous controls, phenotypic characterization, longitudinal sampling, and rich patient metadata are required to (1) identify the characteristic microbial changes involved in EMs and their cause-effect relationships, (2) elaborate deeply the underlying mechanisms contributing to the pathogenesis of EMs, and (3) determine whether the inflammatory environment of EMs is involved in an imbalance of intestinal microbiota. The postulated mechanisms of EMs involvement in gut dysbiosis also deserve deep investigation. Indeed, there are still many challenges to developing gut microbiota-target therapeutics for EMs. Therefore, further exploration and research are needed in the future.

## Ethics statement

Informed consent was obtained from all subjects involved in the study.

## Author contributions

FT: Writing – original draft. MD: Writing – review & editing. CX: Writing – review & editing. RY: Writing – review & editing. XJ: Writing – review & editing. MH: Writing – review & editing. YW: Writing – review & editing. MT: Writing – review & editing. YG: Writing – review & editing. JM: Funding acquisition, Writing –review & editing.
